# A broad cuproptosis landscape in inflammatory bowel disease

**DOI:** 10.3389/fimmu.2022.1031539

**Published:** 2022-11-03

**Authors:** Yuan Chen, Xinfang Li, Ran Sun, Jiamin Ji, Fan Yang, Weiliang Tian, Wu Ji, Qian Huang

**Affiliations:** ^1^ Research Institute of General Surgery, Jinling Hospital, Medical School of Nanjing University, Nanjing, China; ^2^ Research Institute of General Surgery, Jinling Hospital, Southeast University, Nanjing, China

**Keywords:** inflammatory bowel disease, cuproptosis, bioinformatics, immune landscape, molecular docking

## Abstract

**Background:**

Cuproptosis, a genetic process of copper-dependent cell death linked to mitochondria respiration, demonstrates its correlation with inhibiting tumoral angiogenesis and motility. Recent studies have developed systematic bioinformatics frameworks to identify the association of cuproptosis with tumors but any non-neoplastic diseases. Therefore, against the background of an increased incidence of inflammatory bowel disease (IBD), the landscape of cuproptosis regulation in IBD is a critical need to be investigated.

**Methods:**

The differentially expressed cuproptosis-related genes (DECRGs) were identified with human sequencing profiles for four inflammatory digestive disorders. Another four independent IBD datasets from GEO were used as a validation cohort. And experimental mice model provides another validation method. Using single sample gene set enrichment analysis (ssGSEA), receiver operating characteristic (ROC) curve, CIBERSORT, and consensus clustering algorithms, we explored the association between immune score and cuproptosis-related genes, as well as the diagnostic value of these genes. Molecular docking screened potential interaction of IBD drugs with the structural regulator by Autodock Vina.

**Results:**

Cuproptosis-related regulators exhibited extensive differential expression in Crohn’s Disease (CD), Ulcerative Colitis (UC), Celiac Disease (CEL), and IBD-induced cancer (IBD-CA) that share common differential genes (PDHA1, DBT, DLAT, LIAS). The differential expression of DECRGs was reverified in the validated cohort and immunohistochemistry assay. Moreover, the cell signaling pathways and ontology mainly focused on the mitochondrial respiratory function, which was highly enriched in Gene set enrichment analysis (GSEA). According to ssGSEA and ROC, when considering the four regulators, which showed robust association with immune infiltration in IBD, the area under the ROC (AUC) was 0.743. In addition, two clusters of consensus clustering based on the four regulators exhibit different immune phenotypes. According to molecular docking results, methotrexate gained the highest binding affinity to the main chain of key cuproptosis-related regulators compared with the remaining ten drugs.

**Conclusion:**

Cuproptosis-related regulators were widely linked to risk variants, immune cells, immune function, and drug efficacy in IBD. Regulation of cuproptosis may deeply influence the occurrence and development of patients with IBD.

## Introduction

Inflammatory bowel disease (IBD), predominantly ulcerative colitis (UC) and Crohn’s disease (CD), has been widespreadly concerned by both physicians and surgeons in the entire world (1). In the past few years, the acceleration of expenditures clearly indicated that IBD has continuously emerged as a substantial public healthcare challenge in the western world, which mainly includes North America and Europe (2). Given that IBD is a chronic, episodic, and systemic inflammatory condition, several presentations and extraintestinal manifestations have been proposed to be associated with poor quality of life in patients with IBD (3, 4). Moreover, the accompanying comorbidities and complications related to IBD, including metabolic syndrome, short bowel syndrome, fibrotic strictures, celiac disease, and colorectal cancer, are debilitating disease process that mandates a multidisciplinary approach in its management (5–8). It has been reported with respect to the major point of commonalities of pathophysiology between UC and CD, such as genetic variants, immune infiltrations, and dysregulated cell death (9–11). Of these, an aberrant increase in the rate of intestinal epithelial cell (IEC) death underlies instances of extensive epithelial erosion, which is characteristic of several intestinal diseases such as inflammatory bowel disease and infectious colitis (11–13). However, the mechanisms of initial cause and multifactorial etiology were far to understand. Further exploration is under urgent need to determine a new specific genetic variant affecting intestinal epithelium cell death and subsequently intestinal homeostasis, so as to expand molecular and cellular underpinnings in IBD pathogenesis.

Metal-dependent cell death was first discovered in 2012 and later implicated in IBD (14). Ferroptosis, a form of nonprogrammed cell death induced by intracellular ferrum, has made much progress in attenuating intestinal injury related to endoplasmic reticulum (ER) stress and lipid peroxidation (15, 16). However, apart from ferrum, chromium, copper, molybdenum, manganese, and zinc were also defined as essential micronutrients with recommended daily intake for IBD. As a cofactor of a wide range of enzymes involved in metabolic pathways, copper has been maintained at extremely low levels based on concentration gradients, thus blocking intracellular cytotoxicity caused by the accumulation effect (17). Recently, attentions have been brought to copper itself, not copper ionophore that triggered a unique form of cell death termed cuproptosis (18). Together with the engagement of copper ionophores, it is the lipoylated components of mitochondrial respiration that are most important in the signaling course of cuproptosis (18). Moreover, the deletion of key regulators of lipoylation (FDX1 and LIAS) conferred cells’ resistance to cuproptosis, highlighting the functional link between protein lipoylation and cuproptosis (18). In addition, FDX1 finds a special mention among numerous metabolic pathways. These included, but were not limited to tricarboxylic acid (TCA) cycle, cholesterol metabolism, bile acid metabolism, and vitamin D synthesis (19). As a result, a robust link has been proposed between cuproptosis and multiple cancer. However, cuproptosis, which may determine intestinal epithelium cell fate, and its transcriptional regulatory network in IBD have not been revealed prior to this contribution. An urgent need exists for us to uncover the unknown truth of cuproptosis in IBD. While this is promising, little is known about the active site underlying the protease’s activity and how to be blocked by molecules.

FDX1, according to crystal structure termed 3P1M in Protein Data Bank (PDB), was a kind of octameric complex with only two unique subunits (20). Although great similarities in X-ray structures can be found between FDX1 and FDX2, the two mammalian adrenodoxin assume highly specific sequence alignment, and thus specific biochemical pathways (19, 21). The primary ligand, iron-sulfur (Fe-S) cluster binds directly to cysteine of FDX1 in the processing of donation electrons to enzymes (22). The Fe-S clusters had a critical function in stabilizing the structure of FDX1 and were considered as an essential cofactor for the mitochondrial respiratory chain. Therefore, we wonder if IBD medications commonly prescribed can alternate the Fe-S clusters to bind the active sites *via* computational protein-ligand docking.

In the present study, we have deeply dissected the expression of cuproptosis-related regulators derived from published large-scale IBD microarrays and RNA-seq datasets to construct the broad landscapes in IBD. Moreover, we also experimentally confirmed the differentially expressed genes in the UC mice model. Apart from this, immune infiltration, risk model, consensus clustering, and protein-ligand docking were all constructed to demonstrate the gene regulatory network (GRN) of cuproptosis in IBD. An important description of the missing link between IBD and cuproptosis will bring new insights and directions for future studies.

## Materials and methods

### Data screening

Datasets required in this work were located by manual search using the gene expression omnibus (GEO) database (available at http://www.ncbi.nlm.nih.gov/geo). For the cuproptosis landscape in IBD patients, datasets regarding UC (GSE87466), CD (GSE3365), celiac disease (GSE113469), and IBD-related colorectal cancer (GSE3629) have been collected at the initial phase. The datasets with GEO accession numbers of GSE179285, GSE92415, GSE126124, and GSE75214 were considered as validation cohort for IBD in this work, of which the inclusion criteria were detailed as follow. Inclusion criteria: 1. IBD dataset containing both UC and CD patients; 2, The size of specimens was greater than 150; 3. The submitting date for each dataset was within the last five years. For RNA-seq data in transcript levels, extraction and normalization were performed by Perl programming language (version Strawberry-Perl-5.30.0; https://www.perl.org) and R language (version R-4.2.1 https://www.r-project.org) respectively.

### Identification and validation of DEGs

Several bioinformatic packages suitable for R language have been operated on the transcript data in order to visualize differential expressed genes (DEGs). The limma package with a false discovery rate has been deemed to be among the most crucial method of determining DEGs (23). In addition, statistical significance was defined as adjusted P-value less than 0.05 throughout this study. Results are presented using a heatmap based on the pheatmap package. Considering the difference in disorders tightly related to IBD, we use the overlapping genes to explore the cuproptosis landscape in downstream bioinformatics. A Venn diagram of DEGs commonly shared in each of the datasets was identified using the JVENN online (http://jvenn.toulouse.inra.fr/app/index.html) (24). Next, the differential expression of cuproptosis-related genes from another four datasets provided insight to validate the previous result, which used the Wilcoxon test based on the ggpubr package.

### Functional enrichment and gene regulatory networks analysis

Enrichr, a website for geneset enrichment analysis, is meta powerful analytical conductor to reveal the biological functions of overlapping genes from multiple libraries (https://maayanlab.cloud/Enrichr/) (25). Cell signaling pathways, as well as gene ontology, were the top two most important sections to characterize the association between biological mechanisms and interlinked diseases. In this article, we take into consideration four libraries, including KEGG, WikiPathways, Reactome, and GO, to specify the shared function of overlapping genes with a standard metric < 0.05. Moreover, we have located topologically credible miRNAs from the miRTarBase database that tend to interact with our overlapping genes by the NetworkAnalyst platform (https://www.networkanalyst.ca/) (26, 27). At the same time, we also identified a gene-transcription factor network using the JASPAR database based on the NetworkAnalyst platform (28).

### Prognostic signature of immune-related cuproptosis overlapping genes

To construct an immune landscape of the overlapping genes, the single sample gene set enrichment analysis (ssGSEA) algorithm was used to quantify the abundance of immune cells (IMC) and immune functions (IMF) (29). Moreover, the Wilcoxon test was applied for comparison of the fractions of IMC and IMF between IBD patients and healthy participants, in which the filter was defined as 0.05. To go further, it is the determination of the correlation between the overlapping genes and the immune components based on Spearman’s rank correlation coefficient that is the most vital foundation for interpreting and acquiring insights into the cuproptosis-related immune landscape. Lately, a hybrid nomogram that incorporates a calibration curve and consistency index (C-index) was created by the “rms” R package. And the accuracy of the immune-related cuproptosis genes for UC prognosis was evaluated using Receiver operating characteristic (ROC) algorithm analysis.

### Consensus clustering in the cuproptosis overlapping genes

According to the expression profile of the overlapping genes, molecule subtypes screening was obtained from the validation cohort and analyzed with the ClusterPlus R package (30). The cumulative distribution function (CDF), total CDF curve area (delta area), and tracing plot were performed as supplementary information to distinguish the detailed similarity between clusters, while the samples were calculated for 100 times. Furthermore, we then sampled each sample, based on the CIBERSORT analysis, to define an immune score representing the absolute 22 immune cells of different cuproptosis-related clusters within a given dataset degree (31). The barplot and boxplot followed were used to confirm the distinction of IBD risk immune cells between the two clusters. IBD risk genes, derived from the Single Nucleotide Polymorphisms (SNPs) (32), were also utilized to identify the association between cuproptosis-related clustering and IBD pathologies (**Supplementary Table 1**).

### Molecular-ligand docking between IBD drugs and FDX1 protein

The crystal structure of FDX1 protein retrieved from the Protein Data Bank (https://www.rcsb.org) was utilized as the model and the ligands were removed (PDB ID: 3P1M, chain A, G). Due to its significant importance in redox potential, the active site that the ligand Fe-S cluster bind to was used as a control. Computational docking is advantageous for both biomolecular mechanism and drug design in IBD patients, and the AutoDock Vina, in which recommended drugs **Table 1** for IBD are docked and ranked, was used to explore the application of IBD drugs in cuproptosis (33). Prior to testing the molecular docking within the optimized model, the cysteines surrounding the active site of FDX1 were operated as rigid, and the structures of the small molecules were assigned with gasteiger charge and rotatable bonds using the recommended method. Grid box (80 Å × 80 Å × 80 Å) centered at (28.723, 16.464, 15.548) Å were used in the docking experiments by utilizing the AutoDock Vina tools.

**Table 1:** Potent IBD drugs.

Name		Chemical Formula	Molecular Weight	Elemental Analysis	Structure
Aminosalicylic Acids	Sulfasalazine	C18H14N4O5S	398	C, 54.27; H, 3.54; N, 14.06; O, 20.08; S, 8.05	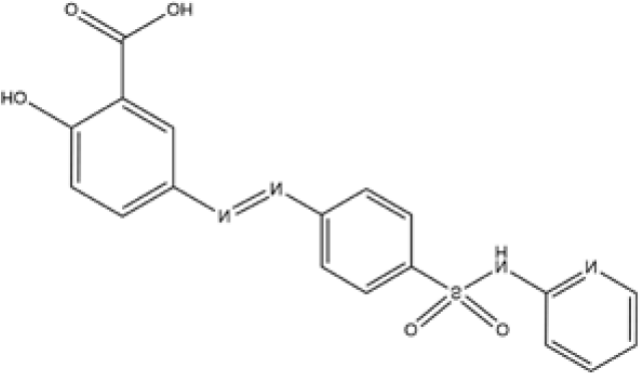
	Olsalazine	C14H10N2O6	302	C, 55.64; H, 3.34; N, 9.27; O, 31.76	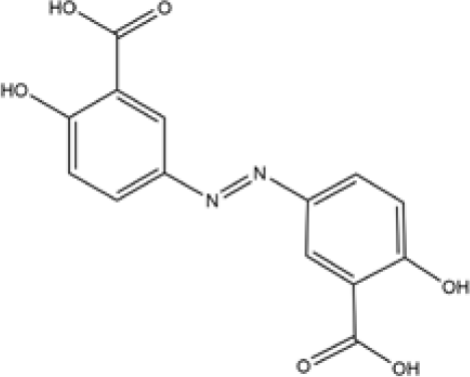
	Mesalazine	C7H7NO3	153	C, 54.90; H, 4.61; N, 9.15; O, 31.34	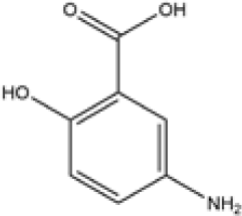
Glucocorticoids	Prednisone	C21H26O5	358	C, 70.37; H, 7.31; O, 22.32	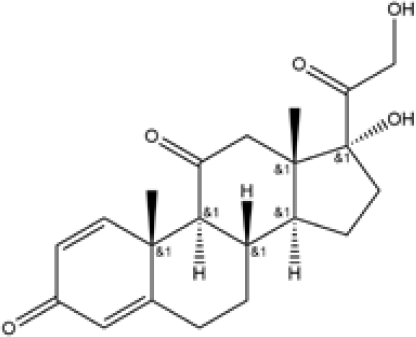
	Hydrocortisone	C21H30O5	362.47	C, 69.59; H, 8.34; O, 22.07	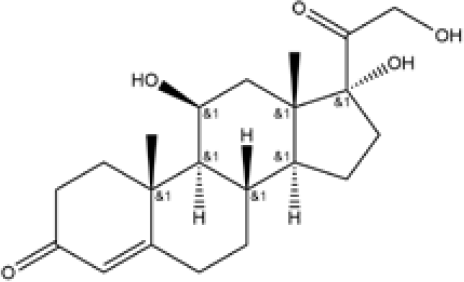
	Budesonide	C25H34O6	430.54	C, 69.74; H, 7.96; O, 22.30	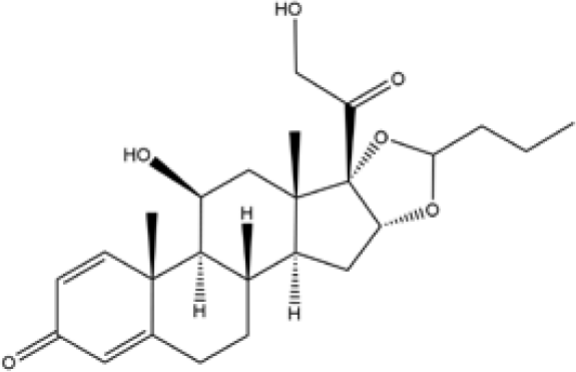
Thiopurines	Azathioprine	C9H7N7O2S	277.26	C, 38.99; H, 2.54; N, 35.36; O, 11.54; S, 11.56	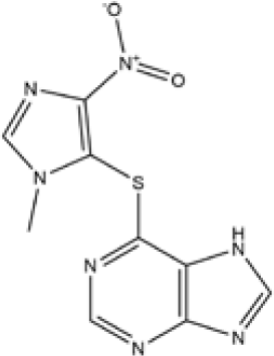
	Mercaptopurine	C5H4N4S	152.18	C, 39.46; H, 2.65; N, 36.82; S, 21.07	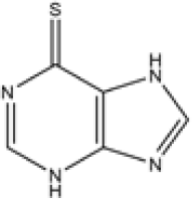
	Methotrexate	C20H22N8O5	454.45	C, 52.86; H, 4.88; N, 24.66; O, 17.60	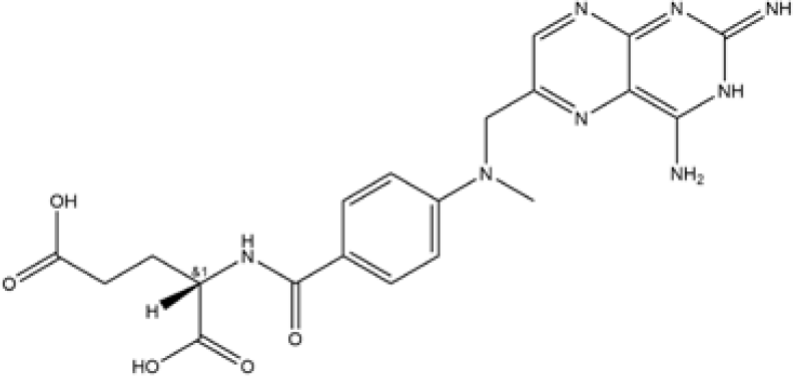
Small Molecule Biologics	Tofacitinib	C16H20N6O	312.38	C, 61.52; H, 6.45; N, 26.90; O, 5.12	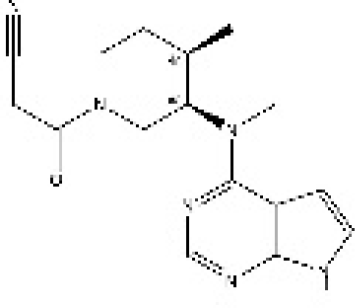
Immunosuppressant	Cyclosporine	C62H111N11O12	1202.64	C, 61.92; H, 9.30; N, 12.81; O, 15.96	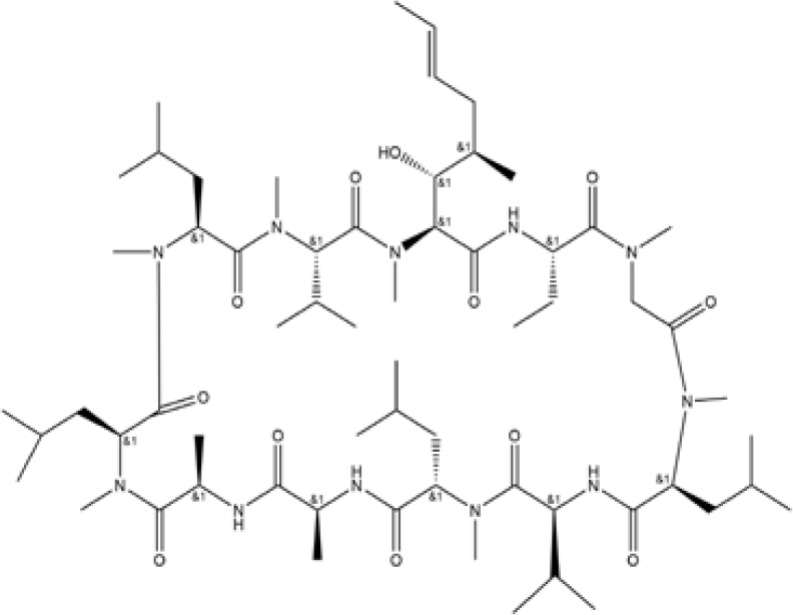

### Experimental UC model and immunohistochemistry

All mice experiments complied with the Guideline for the Care and Use of Laboratory Animals from the National Institutes of Health, USA. C57BL/6 mice (6-8 weeks, 18-22g) were randomly assigned into three groups (n=7): normal control group, ulcerative colitis (UC) group, ulcerative colitis with extra copper (CuSO4·5H2O 7.5mg/kg) supplement (UC-copper) group. We utilized the disease activity index (DAI), including mice’s body weight, stool consistency, and fecal bleeding, which were individually recorded daily to evaluate the success of the UC mice model after induction. Apart from this, further confirmations based on spleen weight and colon length were also conducted. And the inflamed distal colon tissues of both groups were collected, followed by immunohistochemically stained for DBT, PDHA1, LIAS, and DLAT. All statistics were evaluated using an unpaired Student’s t-test for two groups and one-way ANOVA for multiple groups.

## Results

### Genetic relevance between IBD and cuproptosis

**Figure 1:**
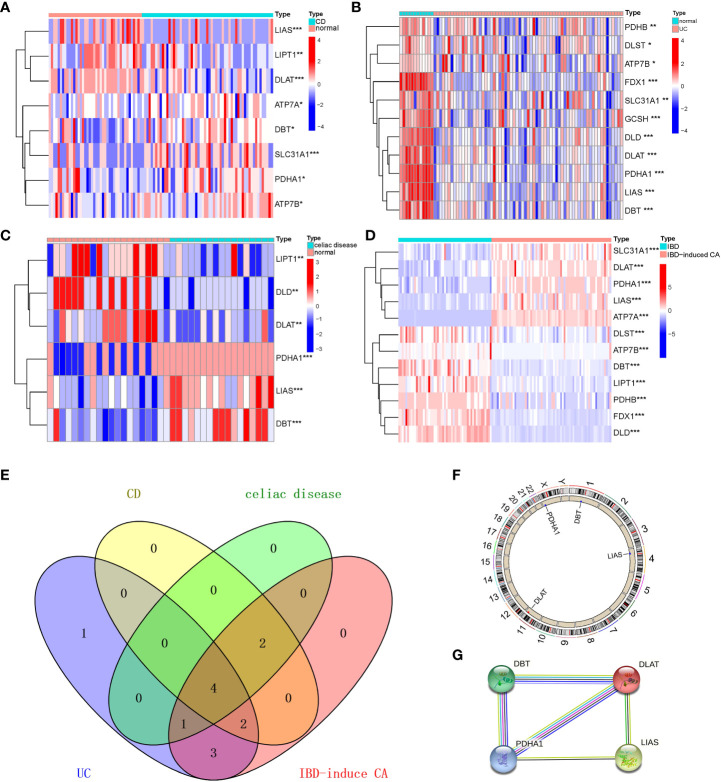
Identification and brief description of cuproptosis-related DEGs shared in Crohn’s Disease (CD), Ulcerative Colitis (UC), Celiac Disease (CEL), and IBD-induced cancer (IBD-CA). **(A–D)** Heatmap showing expression profiles for cuproptosis-related DEGs. Asterisks indicate significances: *p ≤ 0.05, **p ≤ 0.01, ***p ≤ 0.001. **(E)** Venn diagram shows the number of DEGs and intersected DEGs among the conditions including CD, UC, CEL, and IBD-CA. **(F)** Circos diagram reveals the positions of cuproptosis-related overlapping genes in chromosome. **(G)** The protein–protein interaction network shows inner association of these genes.

We have analyzed gene expression regarding cuproptosis from four digestive disorders and found 8, 11, 7, and 12 genes differentially expressed in CD. UC, celiac disease, and colorectal cancer compared with controls, respectively. The DEGs among cuproptosis were described as a heatmap (**Figures 1A–D**) with detailed DEGs expression. The four overlapping genes, including LIAS, DLAT, DBT, and PDHA1, were dysregulated expressed in the interaction between the four digestive diseases using the Venn diagram (**Figure 1E**). Similarly, to achieve a better acceptance of such overlapping genes, the validation cohort, containing four datasets but only IBD patients and healthy control, also revealed significant expression differences of the four overlapping genes (**Figures S1A–D**, and **S2C, D**). However, compared with the discovery cohort, we also detected that 1 out of 5 shared (FDX1, LIAS, DLAT, DBT, and PDHA1) genes between IBD and healthy control was not included in the overlapping genes mentioned above. In addition, an overview of the overlapping genes was visualized briefly by the Circos (**Figure 1F**) and protein-protein interaction (**Figure 1G**) analysis, revealing chromosomal positions and mutual relationships, respectively.

### Functional enrichment and GRN analysis of the overlapping genes

**Figure 2:**
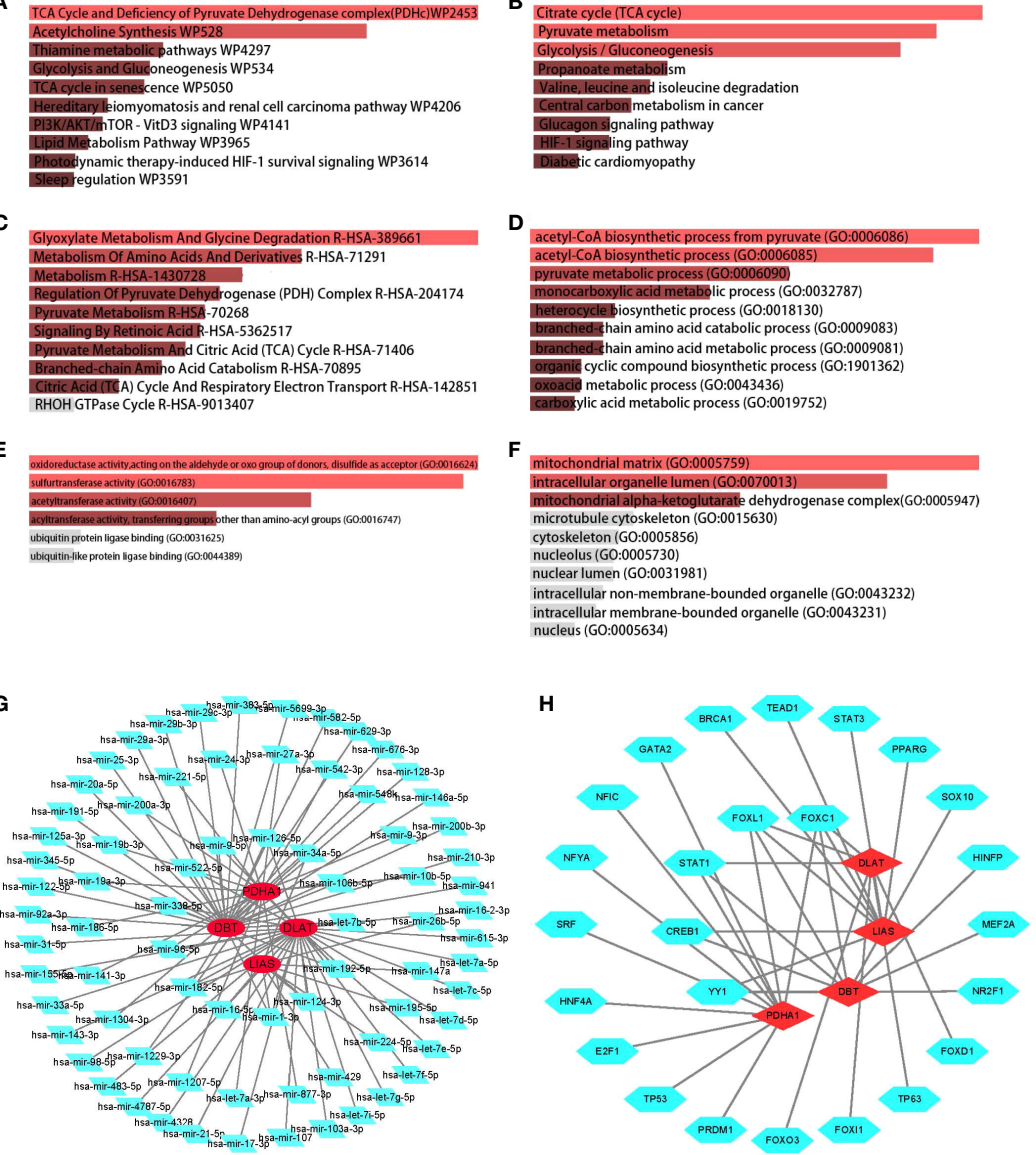
Determination of biological functions and gene regulatory pathway. The bar graphs of enrichment analysis of shared DEGs among CD, UC, CEL, and IBD-CA present that cuproptosis was closely related to mitochondrial respiratory functions. The enriched gene regulatory networks represent the association between genes and regulatory factors, red dots presented in circles represent the genes, and green diamond dots present miRNAs, green polygonal dots present TFs. **(A)** WikiPathway. **(B)** KEGG 2019 human pathway. **(C)** Reactome pathway. **(D)** Biological Processes. **(E)** Molecular Function. **(F)** Cellular Component. **(G)** DEGs-miRNA networks. **(H)** DEGs-TFs networks.

To reveal reliable organism reactions and biological processes to which the overlapping genes contributed, we have merged all the enriched signaling pathways from three libraries commonly used in bioinformatics. It is considered that the most significantly enriched pathway was the Tricarboxylic Acid (TCA) cycle owing to its highest p-value in KEGG and WikiPathway database (**Figures 2A, B**). Furthermore, the metabolism of several substances involved in the TCA cycle also showed significant enrichment, for example, glycolysis and gluconeogenesis, thiamine metabolic pathways, lipid metabolism pathways, pyruvate metabolism, and propanoate metabolism (**Figure 2C**). In addition, the metabolism of amino acids has appeared among highly enriched pathways.

No matter which category it was, the enriched GO terms shared similar metabolic functions that point to the TCA cycle (**Figures 2D–F**). Positive regulation of the TCA cycle was observed particularly in GO cellular components, including mitochondrial matrix, mitochondrial alpha-ketoglutarate dehydrogenase complex, and intracellular organelle lumen. In the case of IBD, the inflammatory response, also termed GO:0006954 in GO biological process (**Figure 2D**), was defined as an essential term to understand the relationship between cuproptosis and the immune landscape.

We then decoded the gene-miRNA (**Figure 2G**) and gene-TF (**Figure 2H**) networks from JASPAR database and optimized them in Cytoscape to reflect the transcriptional changes within the overlapping genes. The green dots of the inner circle ascertained that 5 TFs and 13 miRNAs are associated with more than one overlapping gene, which essentially brings new insights into the interference between cuproptosis-related genes and transcriptional regulators.

### Immune-related cuproptosis landscape in IBD

**Figure 3:**
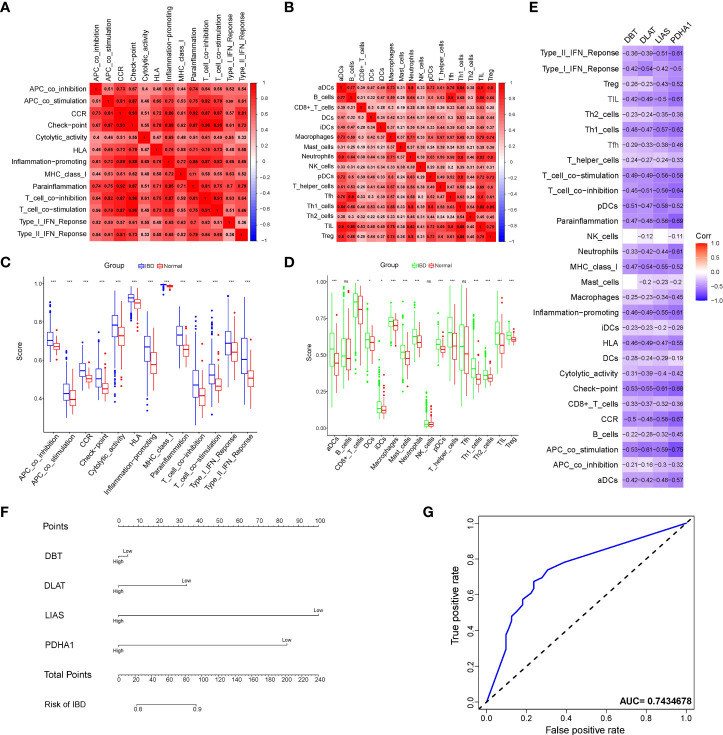
Analysis of immune cell components and diagnostic value of immune-related cuproptosis genes in IBD. **(A, B)** Correlation heatmap of immune components enrichment scores representing both immune cells and immune functions. **(C, D)** Violin plot of differentially immune-infiltrated fraction comparison between IBD and normal control. Asterisks indicate significances: ns no-significance, *p ≤ 0.05, ***p ≤ 0.001. **(E)** Correlation heatmap of the overlapping genes and the immune components. **(F)** Nomogram to predict the diagnostic value of the immune-related cuproptosis genes for IBD. **(G)** ROC curve of the contribution of expression profile regarding immune-related cuproptosis genes to the prediction of IBD.

Immune-related investigations have been screened as integral to understanding IBD, as there was not only an evident risk of immune cell infiltration as a result of the inflammation but also a dysregulated utility of immune function in amplifying the inflammatory response. The heatmap (**Figures 3A, B** and **S3A**), which represented the correlation among IMCs or IMFs in validation cohort after batch correction (**Figures S2A, B**), revealed that the interactions of immune components are involved in the occurrence and development of IBD. The significantly differential immune components are pictured if the Wilcoxon test yields a logarithmic p-value<0.05 between IBD patients and healthy controls in the boxplot (**Figures 3C, D**). More importantly, spearman’s correlation analysis revealed that the overlapping genes were all negatively associated with the differential IMCs and IMFs according to the criterion of moderate association (0.4-0.6) (**Figure 3E**). And the results of differences in immune infiltration after grouping based on expression also confirmed the above point of view (**Figures S3A–D**). Therefore, we used these overlapping immune-related genes to refer to the critical genes regulating copper-mediated IEC death. Considering the aberrant increase in the rate of IEC death, we late described the diagnosis value of the overlapping genes for IBD. Of the genes analyzed, LIAS was the most sensitive indicator for IBD diagnosis in nomogram (**Figures 3F** and **S4**). In addition, Based on the model constructed by the four genes, the AUC was 0.743, suggesting an extremely strong predictive probability for IBD compared to other models (**Figure 3G**).

### Immune and molecular landscape of the cuproptosis subtypes

**Figure 4:**
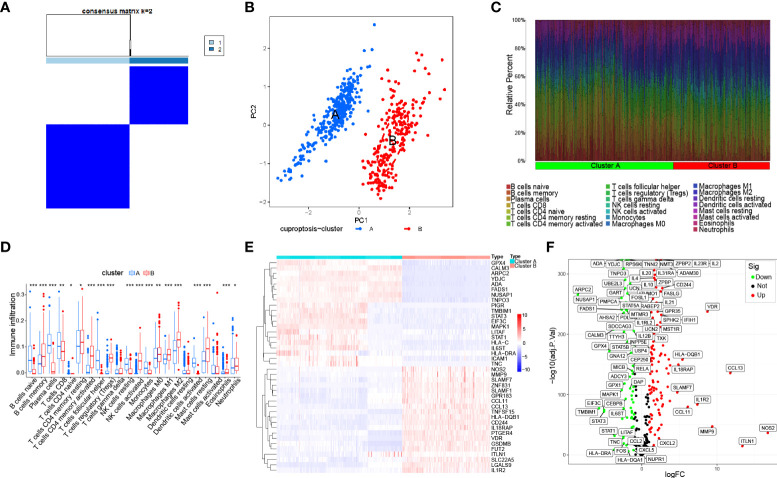
Identification of the molecular subtypes in IBD based on the expression of immune-related cuproptosis genes. **(A)** Consensus matrix (k=2) defining two clusters and their correlation area among 540 IBD samples. **(B)** PCA analysis between cuproptosis cluster A and cluster **(B, C)**. Bar plot visualizing infiltration of 22 immune cells between the two clusters. **(D)** Violin plot of differentially infiltrated immune cells between the two clusters. Asterisks indicate significances: *p ≤ 0.05, **p ≤ 0.01, ***p ≤ 0.001. **(E)** Heatmap showing differential expression of the top 50 genes among 162 IBD risk genes between the two clusters. **(F)** Volcano plot representing all differential IBD risk genes with logFC>1 and P-value <0.05 between the two clusters.

To deeply investigate the divergent regulation of cuproptosis in the IBD pathophysiology, two molecular subtypes were identified based on the expression profile of the constructed model and were also defined in the downstream analysis among IBD patients’ samples (**Figures 4A** and **S5A–C**). The PCA confirmed the apparent distinction between two cuproptosis clusters, which were pictured as two regions with different colored dots, indicating that subclusters of IBD patients showed common genomic cuproptosis-related characteristics (**Figure 4B**). Due to the scarcity of clinical features, it was necessary for us to put the immune infiltration and genetic variants of different clusters first. Different from the above-mentioned immune components, we extracted infiltrated immune cells on the basis of another algorithm called CIBERSORT. The percentage of 22 immune cells in each sample exhibited a different immune condition between the two clusters, even though group differences were not shown clearly in a few immune cells, including T cells gamma delta, dendritic cells resting, and mast cells activated (**Figures 4C, D**). Moreover, the gene expression of IBD risk loci was further retrieved from the validation cohort to probe into the cuproptosis significance in pathogenesis and pathophysiology, and the results revealed that 162 out of 273 genes were differentially expressed between the two cuproptosis clusters, indicating the tight association of cuproptosis and IBD (**Figures 4E, F**).

### The molecular docking landscape on IBD drugs against cuproptosis

**Figure 5:**
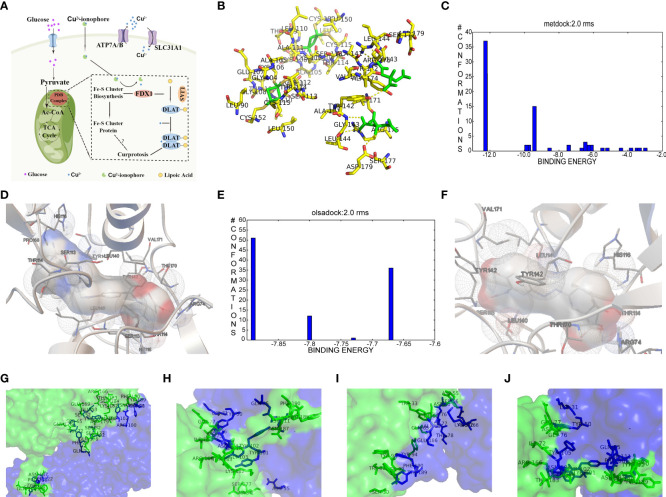
Computational simulation of molecular interaction between FDX1 and IBD drugs. **(A)** Diagram of the regulatory mechanism of cuproptosis. **(B)** Binding region of FDX1 for Fe-S cluster and citrate anion. **(C)** Clusterings of 100 docking results of methotrexate based on binding energy. **(D)** The representative interaction between methotrexate and FDX1. **(E)** Clusterings of 100 docking results of olsalazine based on binding energy. **(F)** The representative interaction between olsalazine and FDX1. **(G-J)** Rigid protein-protein docking images of FDX1 with Infliximab, Golimumab, Usterkinumab, and Natalizumab, respectively.

According to the cuproptosis-regulatory pathway, the most crucial enzyme was, without a doubt, FDX1 (**Figure 5A**). Other than that, the importance of FDX1 in IBD was also seen in the differential expression in the validation cohort. Fe-S cluster and citrate anion, which captured their independence from the crystal structure of FDX1, were used as a control to ascribe the grid box in the docking process (**Figure 5B**). The binding affinity showed interactions formed between FDX1 and IBD drugs from the virtual scanning (**Table 2**). Methotrexate and olsalazine were the first one and the drug with the most occupancy in the top ten conformations, respectively. According to rigid docking results, methotrexate formed 8 hydrogen bonds with arginine(74), threonine(114), histidine(116), tyrosine(142), glutamate(169), and valine(171) (**Figures 5C, D**), and olsalazine formed 6 hydrogen bonds with leucine(140), tyrosine(142), valine(171), arginine(74), and threonine(114) (**Figures 5E, F**). With regard to macromolecular biologics, there were also several hydrogen bonds between FDX1 and agents at the protein surface (**Figures 5G–J**). However, the interactions between binding sites and drugs are not consistent with that of the Fe-S cluster or citrate anion, resulting in the unknown effect of IBD drugs in cuproptosis.

**Table 2:** Docking results of the potent IBD drugs.

	Ligand	BindingAffinity	rmsd/ub	rmsd/lb
1	Methotrexate	-9.8	0	0
2	Methotrexate	-9.6	2.478	1.574
3	Tofacitinib	-9.4	0	0
4	Olsalazine	-9.1	0	0
5	Olsalazine	-8.9	8.449	0.302
6	Tofacitinib	-8.5	8.447	2.21
7	Olsalazine	-8.5	8.4	1.373
8	Olsalazine	-8.4	2.936	2.115
9	Olsalazine	-8.4	2.174	1.745
10	Prednisone	-8.3	0	0
11	Tofacitinib	-8.3	1.888	0.737
12	Olsalazine	-8.2	8.682	2.339
13	Olsalazine	-8.1	8.869	2.201
14	Olsalazine	-8	8.528	1.959
15	Prednisone	-7.8	7.098	1.737
16	Olsalazine	-7.8	8.621	1.87
17	Tofacitinib	-7.7	6.521	2.689
18	Tofacitinib	-7.7	2.355	1.947
19	Tofacitinib	-7.7	8.34	3.501
20	Prednisone	-7.5	7.63	3.268

### Expression of the four genes in UC tissues

**Figure 6:**
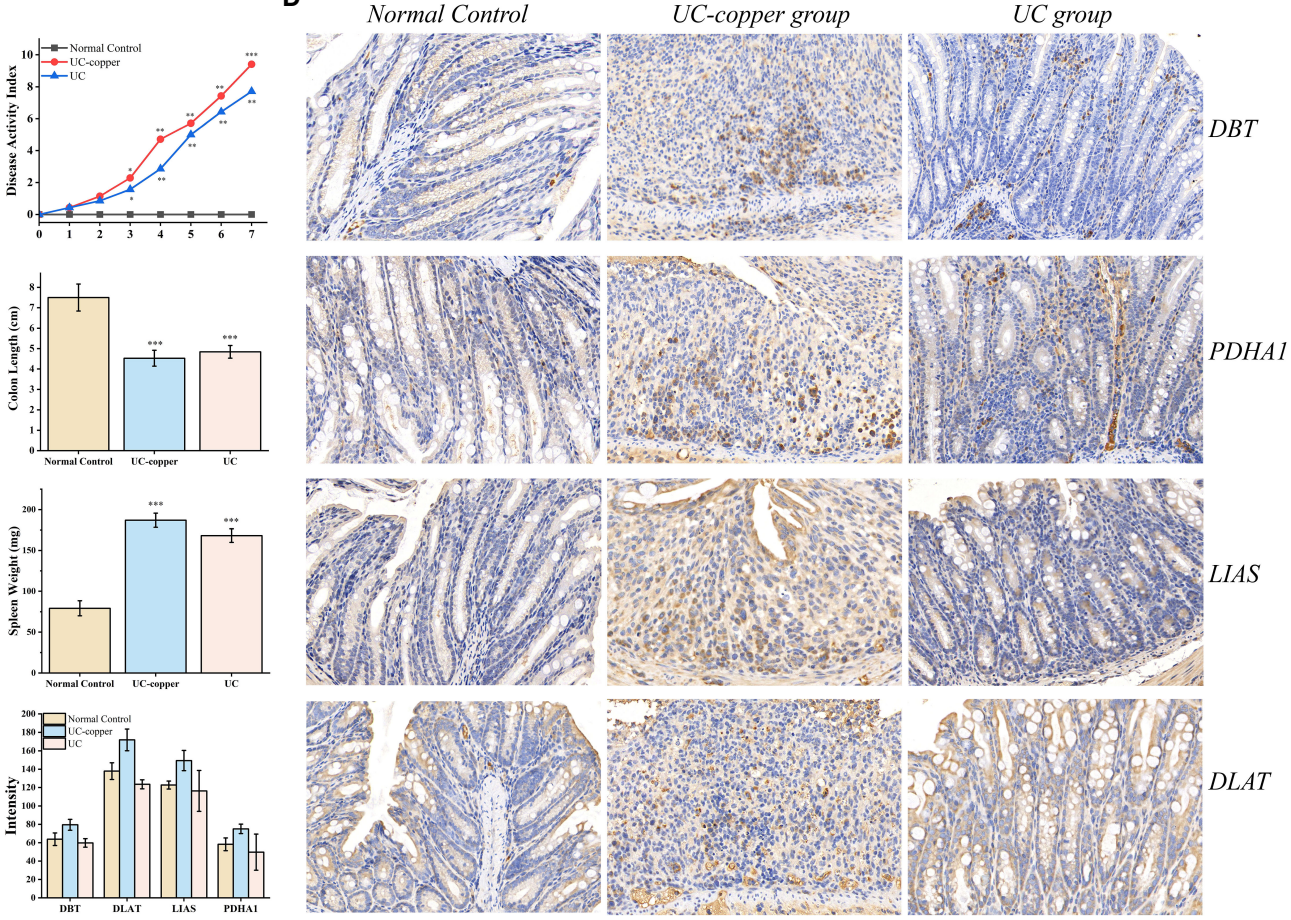
Experimental determination of cuproptosis in DSS-induced UC mice. **(A)** Dynamic changes of the disease activity index at indicated time points. Asterisks indicate significances: *p ≤ 0.05, **p ≤ 0.01, ***p ≤ 0.001. **(B, C)** Differences in spleen weight and colon length between groups. **(D)** Immunohistochemical staining for PDHA1, DBT, DLAT, and LIAS in the distal colonic tissue of each mouse (×20 magnification). **(E)** Comparison of IHC intensity between groups to detect the expression of the four genes.

To quantify the expression of the four genes in IBD, an animal model was constructed by inducing IEC death chemically **Figure 6A**. Colon length and spleen weight confirmed the gross pathological difference between groups, thus identifying the successful establishment of the animal model (**Figures 6B, C** and **S6A, B**). Moreover, we adopted hematoxylin-eosin staining as an alternative method to assess the microscopic colonic injury (**Figures S6C**). Consisted with the results previously described, we detected a slight decrease in expression of the four genes compared with healthy colon tissue (**Figure 6E**). In addition, the strong punctate expression of the four genes in the UC and UC-copper group, which surround multiple nuclei in IHC results, revealed that cuproptosis exert a certain function in the progress of cell death (**Figure 6D**). In conclusion, these results confirmed that the position of cuproptosis was similar to that of ferroptosis UC.

## Discussion

Gastrointestinal epithelium was an absorbing barrier for multi-functional beneficial communities of nutrients, including the metal ions, that regulate both microbial virulence and host immune responses (34). Modern science has yet to determine clinical guidelines for micronutrition intake in IBD patients: the trace element abundances, their balance, and individualized consideration for each patient (35). In the absence of one or more micronutrients, the subsequent management of IBD would become a daunting task for doctors. For example, zinc was an important determinant of immunity and showed a high degree of intracellular concentration variant in its function and responded to infected acquisition and host defense. Conversely, cytotoxic effects would disrupt biological activities while the local zinc concentration was excess (36). Together with the early definition of ferroptosis on the disease course of IBD, we envision a cuproptosis-regulatory method that can make up for the gaps in emerging precision nutrition (15, 16). In this viewpoint, we postulate that the comprehensive landscape of cuproptosis in IBD, based on tremendous advances in genomics and metabolomics, would provide mechanistic insights into pathogenesis to better prevent and manage the disease.

The goal of a comprehensive landscape in cuproptosis with IBD patients was to characterize the shared genes, which acquired from the intersection of CD, UC, celiac disease, and IBD-associated colorectal cancer to describe disease course and improve therapeutic outcomes through the gene expression changes. Our current understanding of cuproptosis highlighted the synergistic effect of FDX1 and LIAS, though with substantial unexplained limitations, which lead to the increased degree of protein lipoylation (18). The certain benefit of LIAS (lipoyl synthase, LipA) was exemplified by lipoyl cofactor biosynthesis (37–39). And the degradation of a 4Fe-4S cluster has led to an altered attachment between the sulfhydryl group and the pendant octanoyl chain (40). As a catalytic substrate of the S-adenosylmethionine (SAM) complex, lipoic acid was taken as an essential redox-active cofactor in numerous metabolic syndromes, one of which was multiple mitochondrial dysfunctions syndrome (41). DLAT (dihydrolipoamide S-acetyltransferase), one of the lipoylated products in cuproptosis, forms a specific insoluble oligomerization while the binding between DLAT and copper was direct (18). The field of genetic variation emerged as an extension of cuproptosis-related analysis to explore the effect of oligomerization in glucose metabolism and of FDX1 deletion on cells’ resistance to copper. The importance of DLAT was seen in mitochondrial respiration as an essential encoding gene of the pyruvate dehydrogenase (PDH) complex (42). A higher expression level of DLAT was associated with lower rates of clinically diagnosed obesity and increased diagnostic risk of illness, including gastric cancer, non-small cell lung cancer induced by PM2.5, infertile, and skin homeostasis (43–47). More importantly, a consistent discovery in the process of mitophagy implicates clearance of impaired mitochondria as a cogent method employed to restrict inflammatory cascade and immune cell homeostasis. With regard to DBT (dihydrolipoamide branched chain transacylase E2) and PDHA1 (pyruvate dehydrogenase E1 subunit alpha 1), they were all mitochondrial enzymes that lipoylated in the process of cuproptosis (18). Given that the dysregulation expression of the two genes had been observed in numerous cancers, it was difficult to relate the two genes to inflammation. The discussion of these cuproptosis-related genes in IBD patients put the dietary copper into a new state. And the specific bioinformatic analyses targeting multiple independent datasets would provide commencement for further exploration of pathogenesis in IBD.

The mucosal immune system, consisting of IEC, microbes, immune cells, and immune mediators, was central to providing a suitable explanation for the occurrence and development of IBD (48). The pathogenesis of cuproptosis in IBD was still undefined but, at its core, its linkage to the immune components was identified, describing an immune landscape of cuproptosis. Our results supported the different abundance of immune cells between healthy control and IBD patients and its tight relationship with cuproptosis-related genes in IBD. In addition to immune cells, immune functions belonging to the immune system were also dominant in early pathologies of IBD. Furthermore, the different clusters exhibited different immune features owing to the biological process of cuproptosis, whereas several signatures of immune cells were similar. The cuproptosis-related immune signature demonstrated the landscape and state of key immune cells, such as T cells, neutrophils, and macrophages. In conclusion, the four genes have been validated to regulate immune homeostasis in IBD and suppress dysregulated immune functions, and the use of immune infiltration as a measurement has been supported.

Inhibition of the targeted enzyme was primarily achieved by simulating binding affinity and bond formation (hydrogen and hydrophobicity) of the most potent IBD drugs (49). Olsalazine, a colonic preparation dependent on bacterial azo reductase, has been approved by the FDA for treating IBD, whether in an acute episode or the relapsing-remitting phase (50, 51). It consists of two 5-aminosalicylic acids connected by an azo bond, resulting in the planar structure. Little evidence of treatment effect in the oral administration of IBD drugs might relate to intestinal epithelium cell death. Olsalazine might have modest success against cuproptosis, in large part because it can bind to FDX1 tightly. Data on docking affinity, binding energy, and hydrogen bond formation are combined to generate hypotheses from which the importance of methotrexate intervention can be raised in cuproptosis. Although the clinical application of methotrexate was limited in IBD, largely to hormone co-administration in moderate to severe IBD, future drug mechanisms and indications will continually improve with cuproptosis (52). These results implied the potential of IBD drugs in the inhibitory effect of cuproptosis.

Currently, the exploration of cuproptosis is in progress, not to mention the effect of cuproptosis on IBD. In this study, we described a comprehensive landscape on the significance of cuproptosis in IBD. Building on the DEGs shared in four datasets, bioinformatic analyses have been employed to show different characteristics of cuproptosis in IBD: functional enrichment, gene regulatory network, immune infiltration, gene-immune components association, diagnostic value, gene clustering, cuproptosis associated SNPs. The current study also presented molecular interaction between FDX1 and IBD drugs to guide future treatment. In conclusion, the viability of the cuproptosis and its ability to change chronic IEC death might have implications for both disease risk and treatment.

## Data availability statement

The datasets presented in this study can be found in online repositories. The names of the repository/repositories and accession number(s) can be found in the article/**Supplementary Material**.

## Ethics statement

The animal study was reviewed and approved by Animal Experiment Ethics Committee of the Jinling Hospital of Nanjing University Medical School.

## Author contributions

YC and XL contributed equally to this work. WJ and QH conceived the idea and designed the experiment. YC and XL conducted experiments and data analysis. YC and XL wrote the manuscript. FY and WT reviewed & editing the manuscript. RS and JJ contributed to the scientific discussion of the article. All authors contributed to the article and approved the submitted version.

## Funding

We thank the National Natural Science Foundation of China (82070579) and the Natural Science Foundation of Jiangsu Province (BE2018712) for their financial support toward this research.

## Conflict of interest

The authors declare that the research was conducted in the absence of any commercial or financial relationships that could be construed as a potential conflict of interest.

## Publisher’s note

All claims expressed in this article are solely those of the authors and do not necessarily represent those of their affiliated organizations, or those of the publisher, the editors and the reviewers. Any product that may be evaluated in this article, or claim that may be made by its manufacturer, is not guaranteed or endorsed by the publisher.
