# Supplementary material for: A broad cuproptosis landscape in inflammatory bowel disease
Source: Front Immunol. 2022 Nov 3;13:1031539. doi: 10.3389/fimmu.2022.1031539 (PMC9669451; doi:10.3389/fimmu.2022.1031539)
Supplement: Supplementary file 1 [file DataSheet_1.docx]

***Supplementary Material***


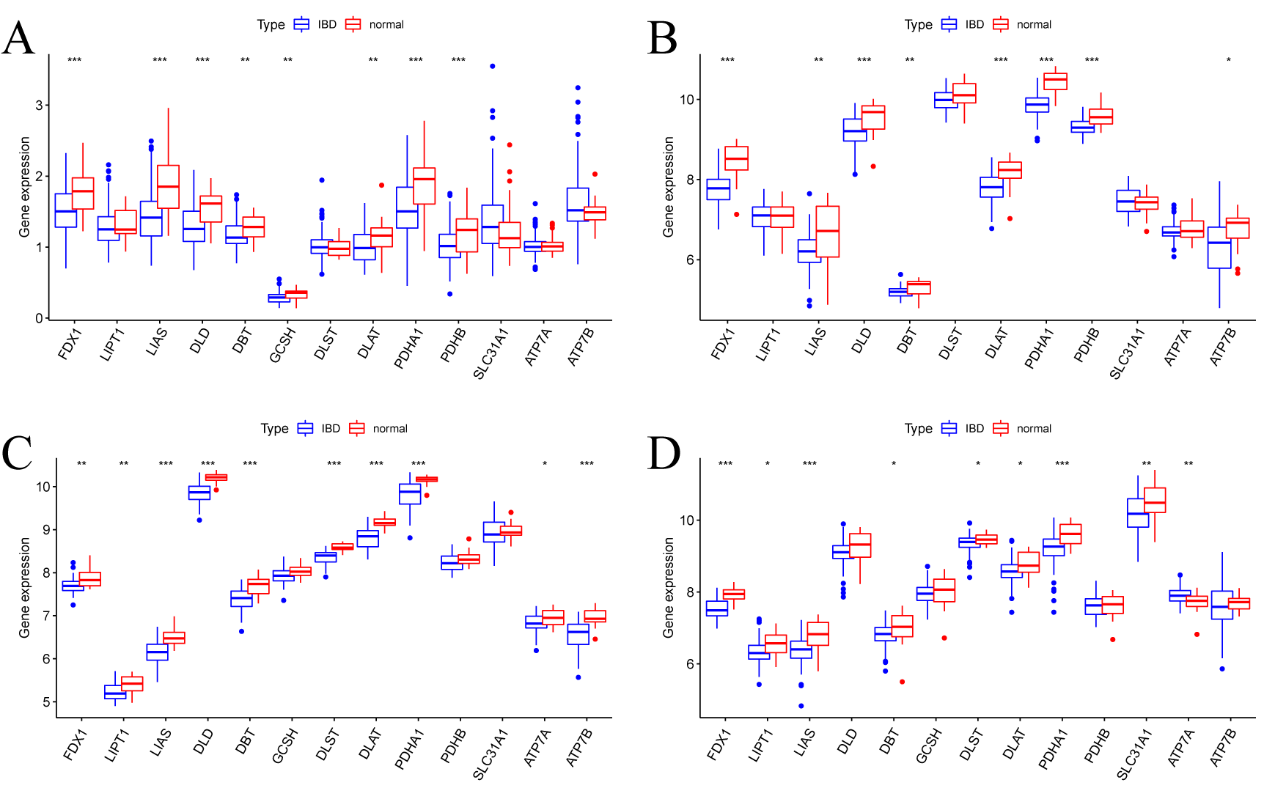


**Supplementary Figure 1**. Boxplots summarizing differential expression analysis in four independent datasets of validation cohort. **A.** GSE179285. **B.** GSE92415. **C.** GSE126124. **D.** GSE75214


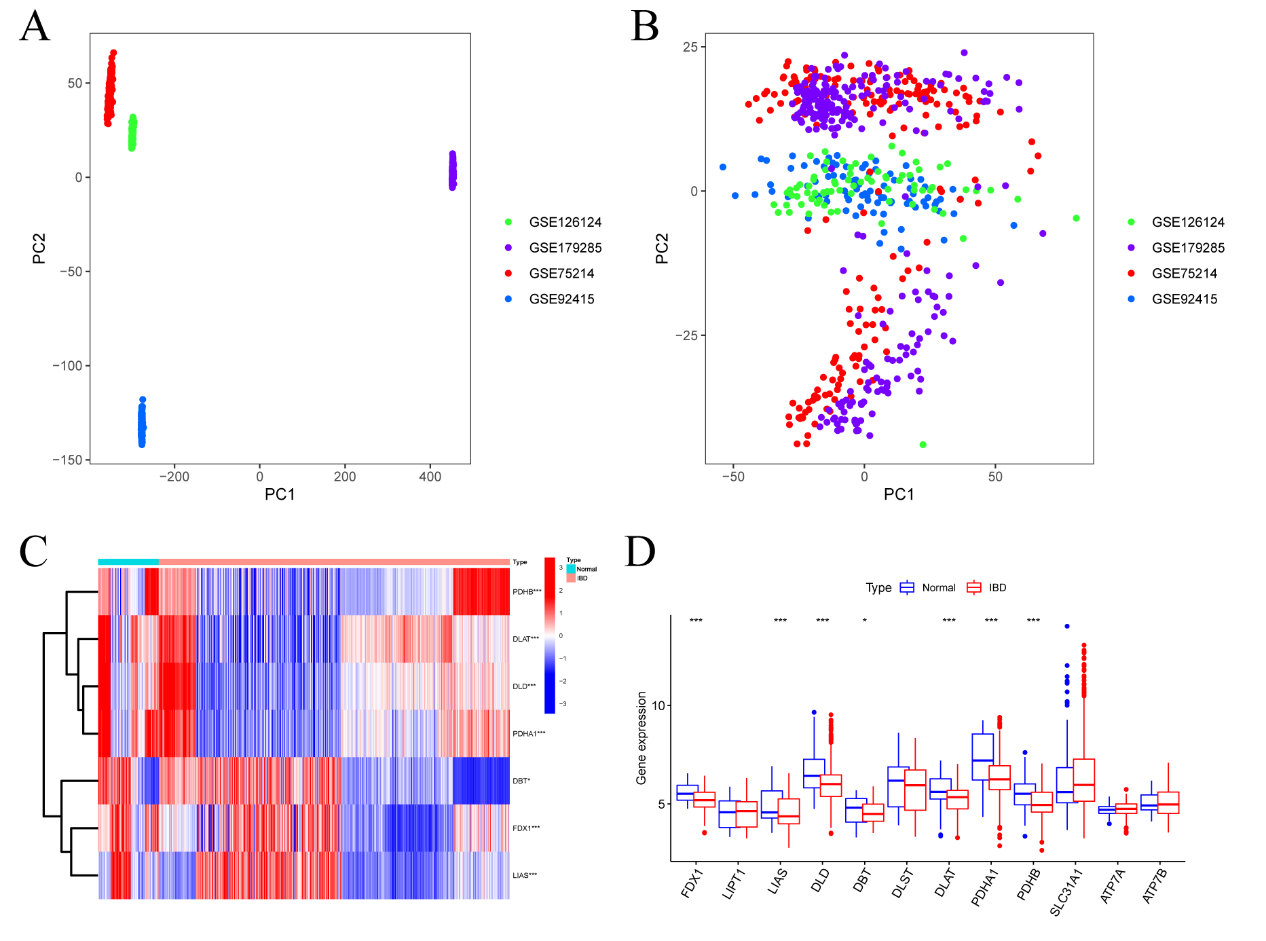


**Supplementary Figure 2**. Batch effect correction for the validation cohort. **A.** Principal components analysis of four independent datasets before correction. **B.** Principal components analysis of consolidated data after eliminating differences. **C.** Heatmap showing expression profiles of consolidated data. **D.** Boxplot showing differences in expression of cuproptosis-related genes.


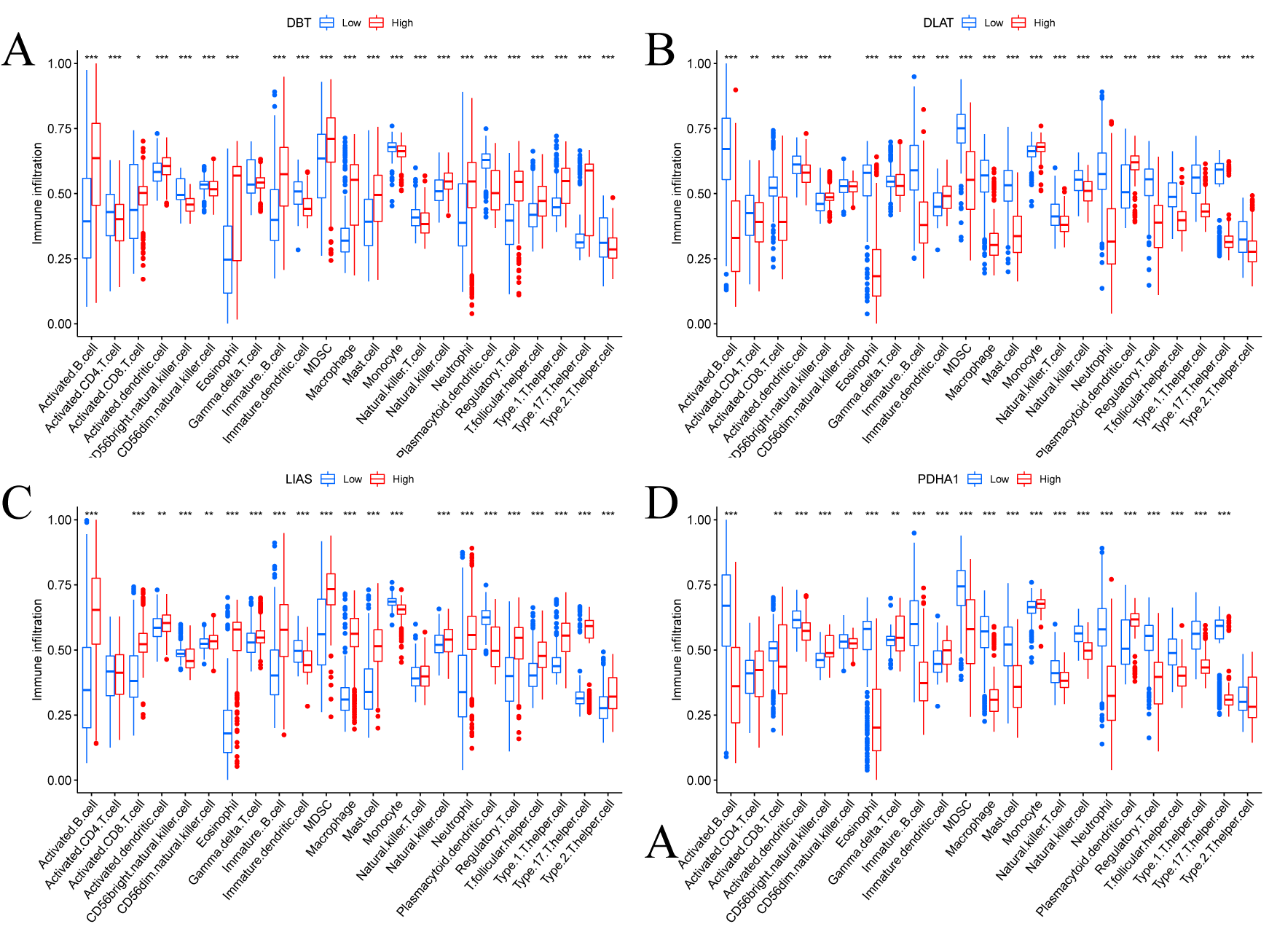


**Supplementary Figure 3**. Differences in immune infiltration grouped based on expression levels. **A.** DBT. **B.** DLAT. **C.** LIAS. **D.** PDHA1


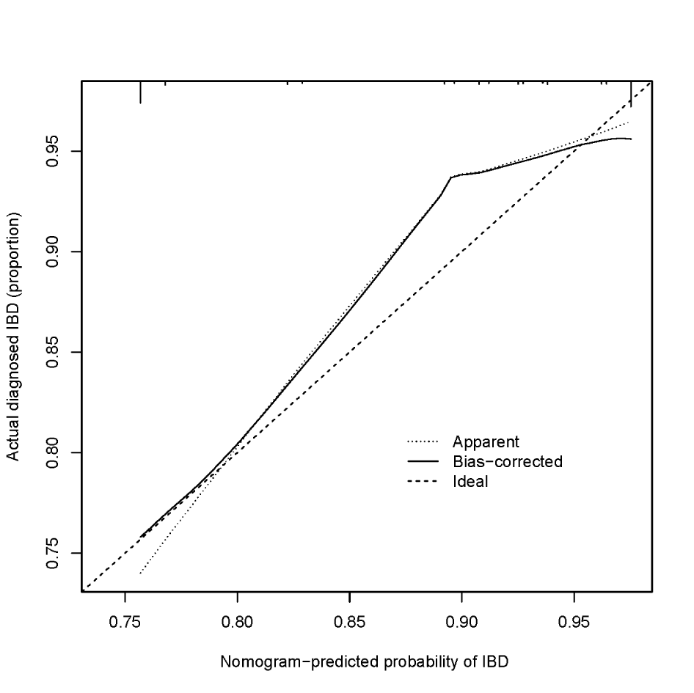


**Supplementary Figure 4**. Calibration curves of the immune-related cuproptosis model to predict the probability of IBD in the consolidated data.


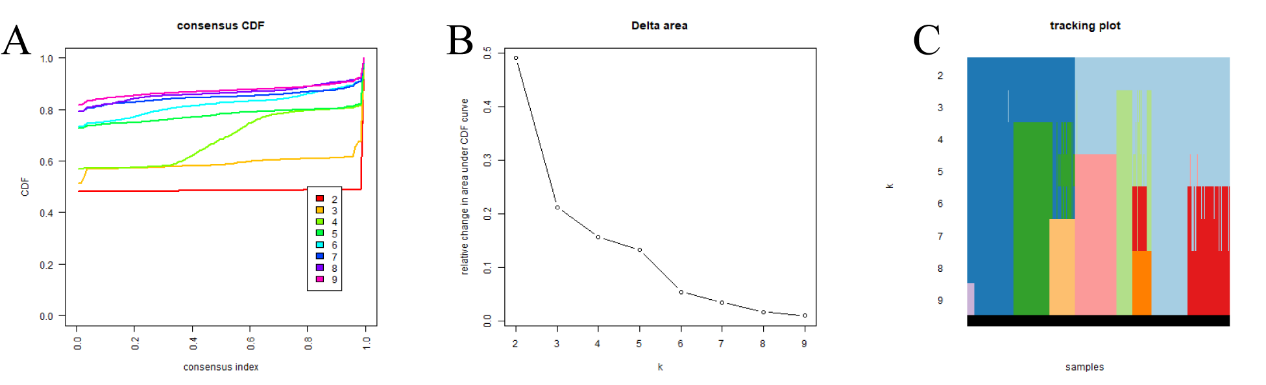


**Supplementary Figure 5**. Identification of consensus clusters based on cuproptosis-related genes. **A.** CDF curve reflecting different cluster numbers **B.** Delta area curve indicating the relative change in area under the CDF curve for each cluster number k compared with k − 1. **C.** Heatmap of sample clustering at consensus k = 2


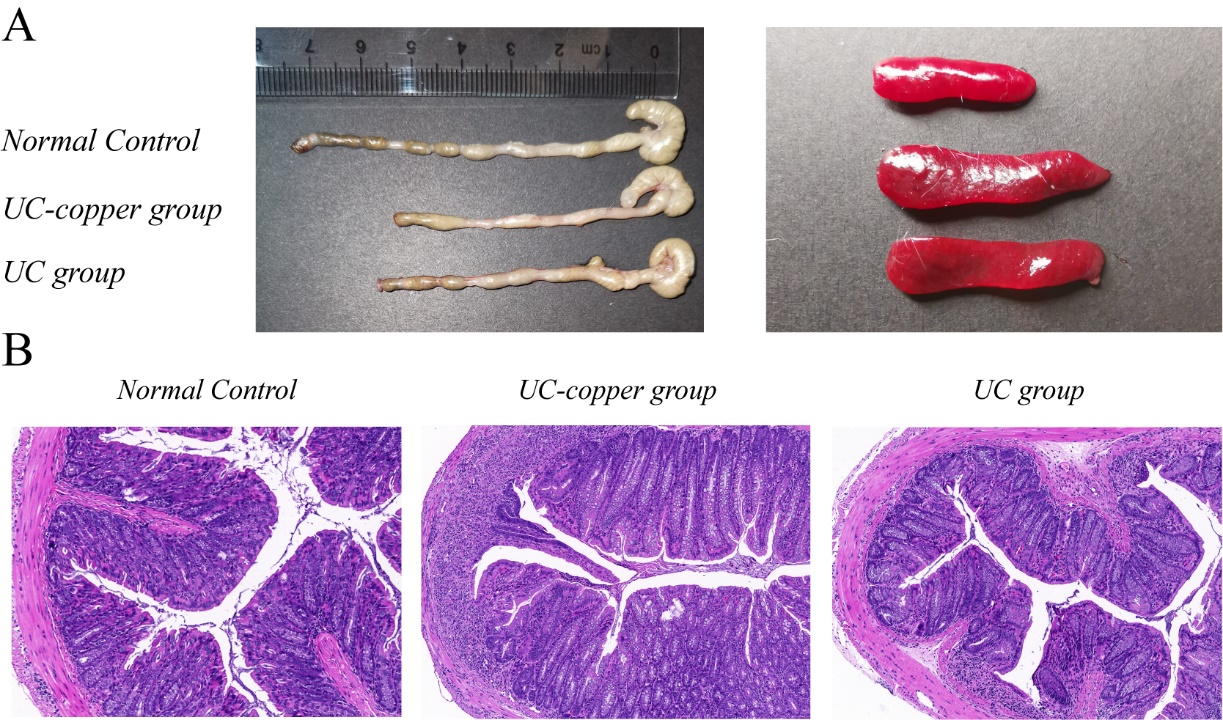


**Supplementary Figure 6**. Determination the extent of inflammatory injury in DSS-induced IBD model. **A.** Measurement of the colon length and spleen weight on the last day. **B.** Assessment of histologically colonic damage based on HE staining.
